# Heterogeneity of Dyscalculia Risk Dependent on the Type of Number Line Estimation Task and the Number Magnitude

**DOI:** 10.3390/ijerph19106164

**Published:** 2022-05-19

**Authors:** Małgorzata Gut, Katarzyna Mańkowska, Jakub Słupczewski, Jacek Matulewski

**Affiliations:** 1 Institute of Psychology, Faculty of Philosophy and Social Sciences, Nicolaus Copernicus University, 87-100 Toruń, Poland; 2 Doctoral School of Social Sciences, Nicolaus Copernicus University, 87-100 Toruń, Poland; km@doktorant.umk.pl (K.M.); 503378@doktorant.umk.pl (J.S.); 3 Department of Informatics, Nicolaus Copernicus University, 87-100 Toruń, Poland; jacekmatulewski@umk.pl

**Keywords:** mental number line, number line estimation, developmental dyscalculia, variability

## Abstract

An ability that is impaired in developmental dyscalculia (DD) is related to number line estimation (NLE). However, due to variability in NLE task performance, group differences do not exemplify the real difficulty level observed in the DD population. Thirty-two of the fifty-two participants posing dyscalculia risk (DR) (mean age = 9.88) experienced difficulties in mathematics. All the children performed two number-to-position tasks and two tasks requiring a verbal estimation of a number indicated on a line, utilizing the ranges 0–100 and 0–1000. The results showed that the estimation error in the verbal task was greater in the DR group than in the typically developed (TD) group for the 0–1000 range. In the number-to-position task, group differences were found for both ranges and the variability within both groups was smaller than it was in the verbal tasks. Analyses of each of the 26 numerical magnitudes revealed a more comprehensive pattern. The majority of the group effects were related to the 0–1000 line. Therefore, considerable data variability, especially in the DD group, suggests this issue must be analyzed carefully in the case of other mathematical capacities. It also critically questions some well-established phenomena and norms in experimental and diagnostic practices.

## 1. Introduction

Developmental dyscalculia (DD) is a clearly defined specific learning disorder [1], which has a neuronal basis (e.g., [2,3,4,5,6]) and leads to the impairment of mathematics skills. This deficit manifests as a difficulty in acquiring several basic mathematical abilities; however, it is not characterized by low intelligence or other developmental, motor, and neurological disorders [7,8]. Children with DD, when compared to those typically developing (TD), show fundamental deficits in number processing skills; for example, they make more errors in counting, numerosity estimation, number naming, number comparison, and the execution of arithmetical procedures. Further, they use immature strategies in mathematical tasks, such as verbal and finger counting and the retrieval of basic arithmetical facts (e.g., [7,9,10,11,12]).

The spatial grounding of numbers, which is the manifestation of their spatial type of mental representation, is based on the metaphor of the mental number line (MNL) [13,14]. It proposes that numbers are represented on a left-to-right continuum. Moreover, numbers and space have a common brain basis in several cortical areas [15,16,17,18,19,20]. Accordingly, DD has been demonstrated to be due to anatomical and functional abnormalities in the regions that are pivotal for basic numerical abilities (e.g., [2,3,4,5,6]). There are several types of spatial numerical associations (SNAs, reviewed by [21,22]) because numerical and spatial information can interact in many different ways. Notably, not all SNAs are correlated with arithmetic skills; even when they are correlated, this relation is not always evident or clear (e.g., [23,24,25,26,27]).

One type of SNA is based on the representation of equidistant relations between numbers and the ability to map numerical intervals on spatial ones. A typical task for the measurement of this SNA category is number line estimation (NLE), in which participants are asked to assess the spatial position of a given number on an empty number line. In some variants, the number line is flanked by the start and end numbers, typically 0 and 100 (or 1000 or even 10,000). Alternatively, there is a number at the beginning of the line and the unit indicating the spatial distance between—for example—0 and 1, which is given as the reference (unbounded number line [28]). For the NLE examination, some researchers use the type of task that requires participants to determine the number value by linking the given mark displayed in a particular locus on the line to the estimated number. In the case of this type of NLE task, participants respond verbally. For example, Ashcraft and Moore [29] examined NLE performance with the use of such a task with children of four different age groups and a group of adults in order to describe the developmental shift from underestimating the numbers located on the left half of the processed number line (which is reflected in the logarithmic pattern of estimation in younger children) toward precisely estimating them as reflected in the linear representation of NLE (in older children and college students). Traditionally, it has been assumed that the ability to position numbers reflects the shape of a numerical representation on the MNL, and the fitting (linear or not) depends on the stage of subjects’ cognitive development or math learning disabilities [29]. The logarithmic pattern of MNL typically observed among younger children has also been found in children with math learning disabilities [30]. The linear representation manifested in precise NLE is interpreted as a clear predictor of later mathematical competencies [31,32,33]. Children with math difficulties present diminished precision in estimating the number position on a number line [30]. The relation between NLE precision and arithmetical skills is stronger for addition and subtraction than it is for multiplication, but the correlation between NLE performance and early arithmetic competencies was determined for the estimation performed on the bounded number line (with the start and end numbers presented [28]; for detailed discussion, see [22]). Previous studies have confirmed this relationship mainly for the number lines ranging from 0 to 100 or 1000. However, poorer precision in NLE being observed in 9–12-year-old children with DD than in the TD group is evident even in the case of NLE tasks with the use of numbers from 1 to 9 [34].

The most common approach to this issue is to focus on the group differences (between children with DD and those who are TD) and analyze descriptive statistics and the fitting to the logarithmic or linear models of NLE in particular age ranges. In the meantime, however, one of the significant factors that complicates the manifestation of symptoms and, consequently, an accurate diagnosis is the considerable heterogeneity of the dyscalculic population. In the literature, reports from studies rarely concentrate on the variability within groups and more or less remarkable differences between particular participants (which are visible in the variability statistics and dispersion of individual results), while they evidently affect the overall result described for groups.

There are several causes of heterogeneity in the DD population. Some authors [35] define the different subtypes of dyscalculia in accordance with prevailing difficulties and brain asymmetry in atypical cortical development. The picture of dyscalculia is further complicated by comorbidities of dyscalculia and several other deficits, such as dyslexia and ADHD (Attention Deficit Hyperactivity Disorder) [7]. In the case of NLE, such variability in the performance of the task influences the group effect in a special additional way because the estimation error may be a result of both underestimation and overestimation. Moreover, the estimation error values may be different in the case of the number range (0–100 vs. 0–1000) and even particular number values. The averaging of such greatly opposite error values may result as very low error magnitudes observed in the whole group, although the individuals revealed clear and pronounced over- or underestimation on the number line.

Von Aster [35] reviewed the state of knowledge concerning the different subtypes of dyscalculia manifesting as the patterns of some impaired numerical competencies that coexist with other abilities, which are preserved in the individuals. The author points out that different types of number representations have distinct brain foundations, which means that different localizations of atypical neuronal development may lead to various types of problems in numerical information processing. Thus, he described neuropsychological categorization, which divides the population by mathematically related deficits into two subtypes [36]. The first is discerned by a low level of both mathematical abilities and reading and spelling, while the second is characterized by poor performance in mathematics but a typical level of reading and spelling abilities. This results in completely different patterns of neuropsychological deficits and scores obtained in each subpopulation for different types of tasks involving various numerical competencies (e.g., spatial–numerical abilities vs. non-symbolic number estimation, symbolic number comparison, or digit naming). Therefore, in the literature, the subtypes of DD are described in relation to the functional disorders of particular brain areas. For example, Geary [37] distinguished different types of DD in relationship to right or left hemisphere dysfunction, defining memory, procedural, and visuo-spatial subtypes. The memory subtype, as a result of left hemisphere dysfunction, is characterized by impaired mathematical fact retrieval and is often correlated with a low level of reading capacities, while the visuo-spatial subtype related to dysfunctions in the right hemisphere manifests in the processing of spatial number representation. Moreover, the localization of the brain foundation of several numerical abilities changes over time. In accordance with some models concerning format-specific brain modules [38,39,40], several authors have described and defined several other types of dyscalculia (calling them “digit dyslexia”, “number–fact dyscalculia”, or “developmental procedural dyscalculia”). For example, with digit dyslexia, children show typical skills related to age in reading and spelling but reveal deficits in reading Arabic numbers or in writing them when they are dictated. Another classification of DD subtypes was proposed by Brandys and Rourke [36], who linked them to educational competencies (not only mathematical capacities but also reading and spelling).

On the basis of the results of his study, von Aster [35], using the battery containing 12 subtests for the examination of several numerical abilities, characterized three types of DD: verbal, Arabic, and pervasive. Skagerlund and Träff [41] showed that the different profiles of numerical ability deficits observed in children resulted from the distinct cognitive origins of DD. The authors examined the aptitude of 77 children for symbolic and non-symbolic number comparison, number naming, and NLE. Importantly, the participants also performed several reading, verbal, and visuo-spatial working memory tasks, as well as the Stroop task. Using these data, the authors tested two alternative hypotheses concerning the cause of DD: the approximate number system hypothesis [13,42] and the access deficit hypothesis [43] to distinguish two subtypes of dyscalculia characterized by different cognitive profiles: arithmetic fact dyscalculia and general dyscalculia. Recently, Kissler et al. [44], analyzing a large sample of children with dyscalculia using the mixture model, identified two subtypes of deficit on the basis of a wide range of data obtained from the measurement of intelligence, reading proficiency, memory, attention, and several mathematical abilities. The authors indicated that two subpopulations of children with DD—slightly and strongly impaired—differ mainly in their profile of mathematical skills; however, the attention level indices seemed to differentiate the DD populations, while intelligence, working memory, and reading fluency did not.

All of these studies made important contributions to both DD diagnosis and participant recruitment to studies on dyscalculia. Moreover, these results provide some idea of how complicated the picture of this deficit is and how many factors affect the pattern of symptoms observed in individuals. One of the most spectacular bodies of evidence on the distinction between the phenomena of spatial–numerical association obtained on the group level and the same examined on the individual level is the analysis result of the data collected from 1105 participants in 17 labs reported by Colling et al. [45]. They undertook the replication of the well-known attentional SNARC (Spatial–Numerical Association of Response Codes) effect [46], which has been considered for almost 20 years as a strong confirmation of the link between numbers and space. The SNARC effect is regarded as the exemplification of horizontally organized MNL [13,19,47], and the attentional SNARC effect is particularly based on the observation that participants are faster in the detection of left-side targets when these stimuli are preceded by low magnitude Arabic numbers (1 or 2) and, similarly, of right-side targets and high magnitude Arabic numbers (8 or 9). The authors of the replication used the datasets (which revealed a clear attentional SNARC effect at the group level) to explore this effect at the level of individual participants. They found that the SNARC effect obtained in the groups came from less than 50% of the participants, who revealed this effect in their reaction times. In many individual cases, the effect was minuscule, absent, or reversed. This means that the idea that some strongly confirmed group-level effects reflect the phenomena (which are present in the majority of the population) must be questioned. Thus, the authors showed that the SNARC effect is often manifested in the individual results of the minority or small subgroups of the population.

We considered that the same might be true for many other phenomena, including some behavioral markers of cognitive deficits (e.g., NLE performance in dyscalculia). Thus, the present study aimed to display a dispersion of the scores obtained within the group of children with dyscalculia risk (DR), as well as those who are TD, focusing on one of the capabilities, namely NLE task performance, which is strongly impaired in dyscalculia. Despite widely proven differences between children (or adults) with dyscalculia and their healthy peers (wherein the former show poorer numerical abilities), the range of dependence of these group differences on the differentiation within both groups is still not clearly demonstrated in the literature or psychological practice. On one hand, in the case of NLE, it is well known that an estimation error is larger in children with dyscalculia than it is in the TD group. On the other hand, our frequent observations of the NLE task performance recorded in relation to the many examined children led us to suggest that the size of the estimation error and its direction (under- or overestimation) truly varied between individuals. As a result, the group differences that can be revealed in the descriptive statistics could be heavily understated. This is because a great percentage of participants might show an overestimation of the locus of particular numbers on the MNL, while the rest of the group would show an underestimation, even in the case of the same numerical magnitudes. As a result, the mean group value may be close to zero; however, it does not, in fact, mean that this group of participants is characterized by high precision in their NLE ability. Thus, it is worth emphasizing that a measurement of several basic numerical capacities, during both scientific investigations and the clinical diagnosis process, usually requires focusing on descriptive statistics and analyzing group differences, whereas it is equally important or even more meaningful to look at individual data variability as well as to investigate how the estimation error level depends on the number range (here, we used 0–100 and 0–1000 number lines), the particular number magnitude, and the type of NLE task (the type of stimulus and response). This could have valuable implications for dyscalculia screening procedures, as well as the planning of educational or therapeutic interventions based on MNL processing.

We considered that the same might be true for many other phenomena, including some behavioral markers of cognitive deficits (e.g., NLE performance in dyscalculia). Thus, the present study aimed to display a dispersion of scores obtained within the group of children with dyscalculia risk (DR), as well as those who are TD, focusing on one of the capabilities, namely NLE task performance, which is strongly impaired in dyscalculia. Despite widely proven differences between children (or adults) with dyscalculia and their healthy peers (wherein the former show poorer numerical abilities), the range of dependence of these group differences on the differentiation within both groups is still not clearly demonstrated in the literature or psychological practice. On one hand, in the case of NLE, it is well known that an estimation error is larger in children with dyscalculia than it is in the TD group. On the other hand, our frequent observations of the NLE task performance recorded in relation to the many examined children led us to suggest that the size of the estimation error and its direction (under- or overestimation) truly varied between individuals. As a result, the group differences that can be revealed in the descriptive statistics could be heavily understated. This is because a great percentage of participants might show an overestimation of the locus of particular numbers on the MNL, while the rest of the group would show an underestimation, even in the case of the same numerical magnitudes. As a result, the mean group value may be close to zero; however, it does not, in fact, mean that this group of participants is characterized by high precision in their NLE ability. Thus, it is worth emphasizing that a measurement of several basic numerical capacities, during both scientific investigations and the clinical diagnosis process, usually requires focusing on descriptive statistics and analyzing group differences, whereas it is equally important or even more meaningful to look at individual data variability as well as to investigate how the estimation error level depends on the number range (here, we used 0–100 and 0–1000 number lines), the particular number magnitude, and the type of NLE task (the type of stimulus and response). This could have valuable implications for dyscalculia screening procedures, as well as the planning of the educational or therapeutic interventions based on MNL processing.

The aim of this study is not to confirm any of the theories described in previous studies, which were mentioned above, or to indicate some cognitive markers, which could help in defining the dyscalculia subtypes in our study sample. Our goal is rather to shed light on the issue of considerable variance found within the groups, especially with reference to DR (because of the representatives of the different subtypes, as well as the impact of many factors on the symptom profiles—e.g., attention deficits), which is undervalued in the literature. Thus, our general questions were as follows: 1. What do we know about the differences between children with dyscalculia and healthy controls until we explore the examined variables on the individual level? and 2. To what extent does this variability blur the real picture of these group differences between deficit and typical development? Moreover, we supposed the group differences as well as individual variability within the groups would depend on the following: type of task, range of number values. If so, it provokes questions regarding the types of tasks that should be chosen for the process of examining and diagnosing DD or DR, particularly the latter.

## 2. Materials and Methods

### 2.1. Participants

A total of 52 children (26 girls and 26 boys, mean age = 9.88, SD = 0.76, ranged from 8 to 11) participated in the study, out of which 32 had difficulties in mathematics diagnosed as dyscalculia risk (DR group, 14 girls and 18 boys, mean age = 9.91, SD = 0.78). Five participants were left-handed. They were compared to 20 typically developed children (TD, control group) with normal achievement in mathematics (12 girls and 8 boys, mean age = 9.85, SD = 0.75). The mean ages did not differ significantly between the DR and TD groups (*Z* = 0.33; *p* = 0.74), nor did the number of boys and girls (χ^2^(1) = 0.03; *p* = 0.86).

The participants were students of the elementary schools from seven voivodeships of Poland. They all received regular education in mathematics. Participants were recruited to the DR group in two ways: (1) on the basis of the results obtained in two batteries used in Poland for dyscalculia risk assessment (which were performed within the present study): computerized scale “Prokalkulia 6–9” [48] and paper–pencil scale “Skala Gotowości Matematycznej i Ryzyka Dyskalkulii” [49], or (2) by psychologists in psychological and pedagogical guidance service and counseling centers, on the basis of their standard diagnostic criteria, including, for example, general intelligence within a normal range, low score (under 8 points) in the Arithmetic subtest of the Wechsler Intelligence Scale for Children-Revised (WISC-R; [50]), the level of mathematical achievement at school, and the psychologist’s, as well as parents’, observations. Importantly, DR participants had no diagnosis of dyscalculia because, in Poland, this deficit may be diagnosed quite late, at the end of primary school time. Thus, DR participants of this study were classified as children with dyscalculia risk instead of the dyscalculia group. Inclusion criteria for all children (DR and TD) were as follows: at least normal IQ and no history of neurologic or psychiatric disorder.

The parents of all participants gave their informed consent to participate. The procedures used in the study were approved by the local ethics committee of Nicolaus Copernicus University in Toruń. All participants were healthy and had normal or corrected-to-normal vision.

### 2.2. Tasks and Stimuli

**Figure 1:**
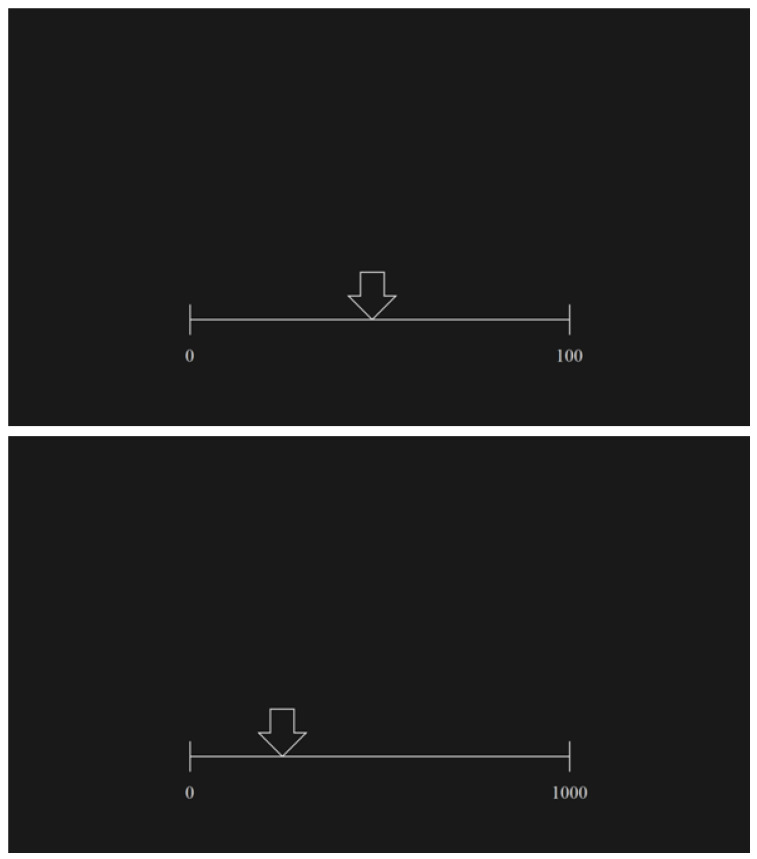
Examples of stimuli used in the tasks that required verbal estimation of the number magnitude pointed by an arrow on the empty number line: 0–100 (**top**) and 0–1000 (**bottom**).

The study paradigm contained four tasks. The first two tasks were based on the verbal determination of the numbers, which were pointed by an indicator (a white arrow) placed on the empty number line that was flanked by two marginal numbers (Figure 1).

These two tasks were divided into two ranges: from 0 to 100 and from 0 to 1000. The participants were asked to respond verbally to a researcher’s question: “Please tell me, what number from the numerical interval 0–100 (0–1000) is located here in the place that is indicated by an arrow?”. The children were required to determine the number as precisely as possible. There was no time pressure during these tasks. The verbal answers of the participants were recorded and noted.

**Figure 2:**
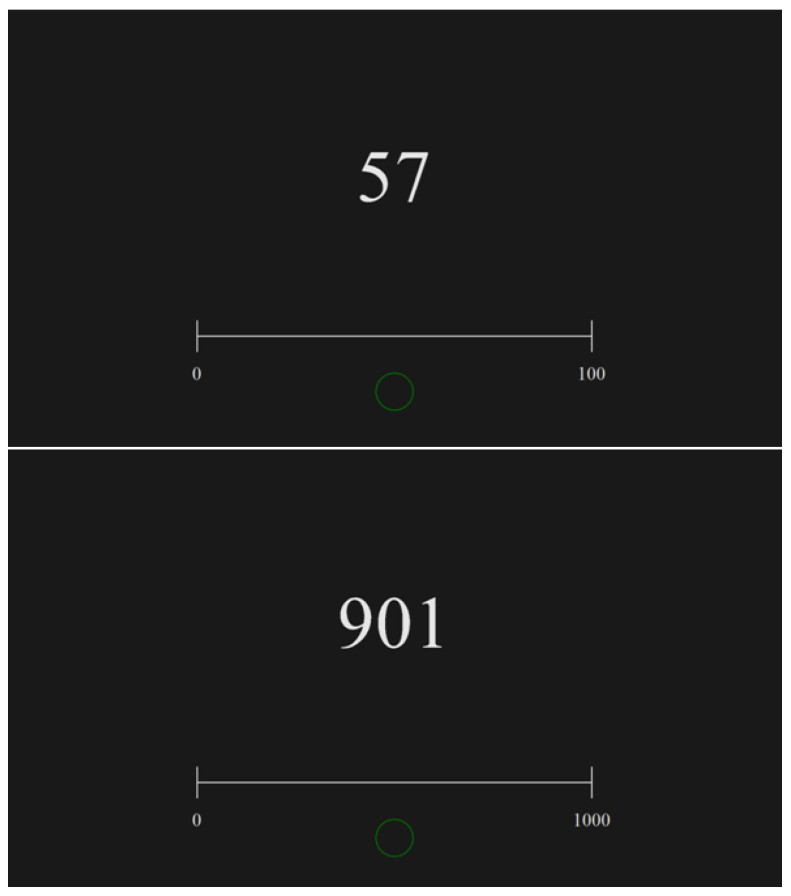
Examples of stimuli used in the number-to-position tasks that required estimating the location of the given number by pressing an estimated place on the screen, with the use of two numerical intervals: 0–100 (**top**) and 0–1000 (**bottom**).

In the following two tasks, children were asked to place the number (which was presented on the screen above the line; see Figure 2) by touching the point on the empty number line where the participants assessed the locus of it. In task 3, the participants estimated the locations of numbers on the line flanked by 0 and 100, and, in task 4, the number range was from 0 to 1000. The instruction was as follows: “Please indicate the place on this number line that is referred to this number displayed above the line. You can do it by pressing a screen in this particular point of the line with your index finger, and then you have to confirm your choice by pressing this green circle below the line.” Again, children were required to determine the location of the number as precisely as possible, and there was no time pressure during these tasks.

The numbers used as stimuli in tasks 1 and 3 were as follows: 3, 4, 6, 8, 12, 14, 17, 18, 21, 24, 25, 29, 33, 39, 42, 48, 52, 57, 61, 64, 72, 79, 81, 84, 90, and 96, while, in tasks 2 and 4, there were the following 26 numbers: 31, 44, 62, 89, 123, 143, 176, 182, 215, 243, 253, 297, 333, 395, 421, 489, 526, 577, 610, 644, 724, 791, 814, 847, 901, and 966. Thus, each number used in each task was repeated once (which gives 26 trials in each task). The selection of such particular numbers and their repetitions was based on a study reported by Ashcraft and Moore [29]. They investigated the profiles of MNL 0–100 and 0–1000 using of the same set of numbers (see also [49], who justified the oversampling of number magnitudes in the lower half of the number lines).

The order of the tasks was not randomized. Each participant first performed two tasks with the verbal discrimination of the indicated locus of the number and then the tasks with the pointing of the location of the displayed number. Moreover, children first performed the task with a 0–100 number line (tasks 1 and 3) and then the task with a 0–1000 number line (tasks 2 and 4), thereby completing the simpler task first. The order of numbers used in all tasks was randomized across the trials (displayed number or arrow position were not presented, e.g., in ascending or descending order within the task), and they were also randomized across the participants.

In all four tasks, each stimulus consisted of a horizontal empty number line without ticks marking integer numbers (except the start and end points, which were labeled “0” and “100”, or “1000”, respectively). The line was half of the screen width. The number line was horizontally aligned to the center of the screen and located at 3/4 of the screen height from the top.

In each trial, the number line, an arrow in tasks 1 and 2, and the number presented above the line in tasks 3 and 4, was displayed in white (RGB 230, 230, 230). All stimuli were presented on a black background (RGB 25, 25, 25). The contrast of both colors was reduced to avoid eye fatigue and afterimage effects. The height of each number in the stimulus in tasks 3 and 4 was 222 px, and the width depended on the number value.

The participants were asked to estimate the location of a number on the presented number line (number-to-position NLE task) in tasks 3 and 4 by pointing to the right number position using their right index finger.

There was no time limit for performing all tasks, so children were instructed to determine the number placed in the indicated location (in tasks 1 and 2) and to estimate the location of a given number (in tasks 3 and 4) as precisely as they could. The estimation error value in tasks 1 and 2 was calculated as the result of subtraction: the number magnitude indicated verbally by the participant minus the number that was exactly located in the locus pointed by the arrow. The estimation error in tasks 3 and 4 was calculated as the ratio of the absolute number of pixels between the indicated and correct positions to the length of the whole number line (also measured in pixels), multiplied by 100, and was expressed as a percentage.

For each stimulus, in tasks 1 and 2, an arrow (with a height equal to 1/9 of screen height) was presented directly above the line and pointed to the particular number position. In tasks 3 and 4, the given numbers were displayed above the line at 4/10 of the screen height from the top and the middle of the line.

### 2.3. Apparatus and Software

The participants were comfortably seated at about 60 cm in front of a screen. The stimuli were presented on a 27″ LED touch monitor Iiyama ProLite T2735MSC (Iiyama, Tokio, Japan) with a resolution of 1920 × 1080 pixels. The stimulus presentation and the recording of participants’ responses were controlled by the Asus (Asus TeK Computer Inc., Taipei, Taiwan) VivoBook S15 S510U laptop (Windows 10 Home, Intel Core i5 processor). All tasks were prepared in the Microsoft Visual Studio 2017 Enterprise (Microsoft, Redmond, WA, USA) integrated development environment using C# language. It requires NET Framework 4 Client Profile and Windows 10 (Microsoft, Redmond, WA, USA).

### 2.4. Statistical Analysis

Because data were not normally distributed and the groups were unequal, an appropriate non-parametric test was used to investigate general group differences in NLE error. Group (DR/TD) was considered the between-subjects variable. All statistical analyses were performed using IBM SPSS Statistics v.27 (IBM, Armonk, NY, USA). To examine the group differences (DR vs. TD) for mean estimation error (EE) manifested as the numerical distance between the value marked by an arrow and the value verbally stated by the participant, the data from tasks 1 and 2 were submitted to a non-parametric Mann–Whitney U test for independent samples. Moreover, to examine the effect of the group on mean EE in the number-to-position tasks (tasks 3 and 4), the data were submitted to a non-parametric Mann–Whitney U test for independent samples.

## 3. Results

### 3.1. Effect of the Group on Mean Estimation Error Values in the Verbal Determination of Number Magnitude Indicated on the Number Line

**Figure 3:**
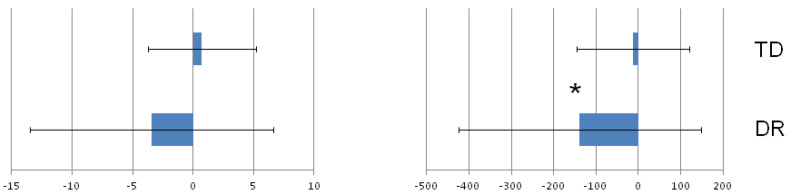
Estimation error (EE; median values represented by bars) obtained in the DR and TD groups in the verbal task with the use of 0–100 number line (**left**) and 0–1000 number line (**right**). The error bars represent the interquartile range (IQR) values. * *p* < 0.05.

In the first step of analyses, the mean group Estimation Errors (EEs, calculated as mean values from all 26 numbers in each group) were compared. The analysis revealed that there was a significant effect of group on EE in the case of the 0–1000 number line (see Figure 3), while the group difference for the 0–100 number line was not statistically significant. The median and Interquartile Range (IQR) values obtained in each group are presented in Table 1.

### 3.2. Group Differences for Estimation Error in the Number-to-Position Tasks

**Figure 4:**
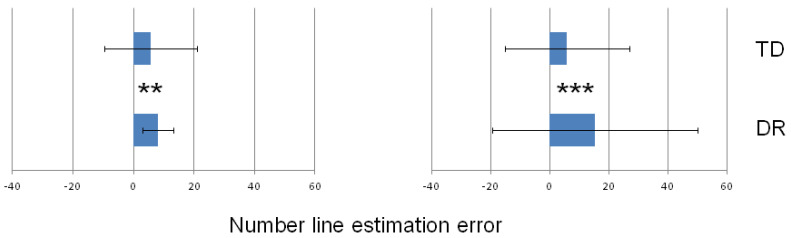
Estimation error (EE; median values represented by bars) obtained in the DR and TD groups in the number-to-position task with the use of 0–100 number line (**left**) and 0–1000 number line (**right**). The error bars represent the interquartile range (IQR) values. ** *p* < 0.01; *** *p* < 0.001.

The mean values calculated for all the number magnitudes employed in the particular task (0–100 and 0–1000, respectively) were used to compare the two groups of participants. Table 2 shows the descriptive statistics (median and IQR values) obtained in each group. A significant effect of the group was found both for the 0–100 and 0–1000 number lines. The estimation error was significantly greater in the DR group than in the TD group in both number ranges. The significant differences between the groups are presented in Figure 4.

### 3.3. Estimation Error Values Calculated for Each Number Magnitude in Tasks 1 and 2

The next step of comparisons for tasks 1 and 2 was focused on the differences between the groups calculated for each number magnitude separately. In the case of the 0–100 number line (task 1), the analyses revealed significant differences between the groups for the following number magnitudes: number 18 and number 33. The descriptive statistics obtained for each number magnitude used in 0–100 NLE are presented in Table 3.

In the case of the 0–1000 number line, the analyses revealed significant differences for many more numerical values. Importantly, in the case of number 31 (number line 0–1000, task 2), the EE value obtained by one participant in the TD group was excluded from analysis because the EE recorded for that particular numerical value in the case of that individual was strongly distant from the EEs obtained for other numbers, as well as from the results of the rest of the participants (this particular individual EE value exceeded 20 SD).

Significant differences for the range 0–1000 were found for the following number magnitudes: 44, 62, 123, 176, 182, 215, 243, 253, 297, 333, 421, 577, and 610.

The differences between the groups with respect to the remaining number magnitudes were insignificant. The descriptive statistics obtained for each number magnitude used in the 0–1000 NLE are presented in Table 4.

### 3.4. Data Variability within Groups in NLE Error

The mean EEs calculated for the individual participants on the basis of all the trials in the task (all 26 numbers used in task 1 and task 2, respectively) may be markedly different from participant to participant in regard to the size of EE, as well as the direction of this estimation error (underestimation or overestimation). This means that the EE value may be positive when the participant underestimates the localization of a certain number or negative in the case of overestimation. The same may be observed in the case of the mean EEs calculated separately for each singular number magnitude.

**Figure 5:**
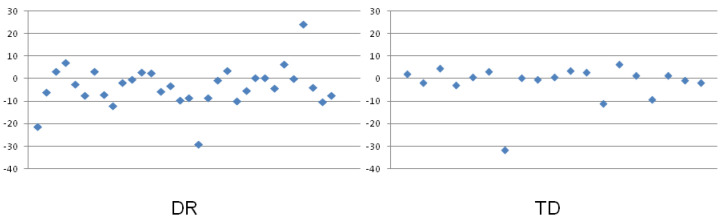
Scatter plots showing the mean EEs for all numbers used in the 0–100 verbal task calculated for each participant. The results obtained in the DR group are presented on the (**left**) and the results obtained in the TD group are on the (**right**).

**Figure 6:**
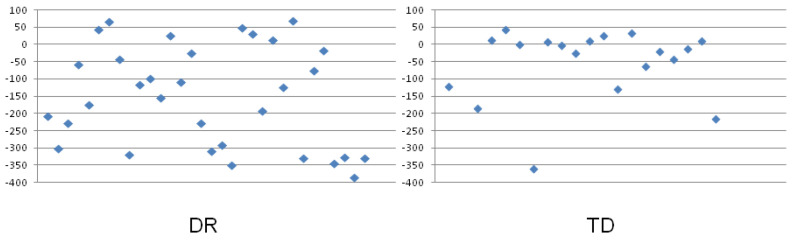
Scatter plots showing the mean EEs for all numbers used in the 0–1000 verbal task calculated for each participant. The results obtained in the DR group are presented on the (**left**), and the results obtained in the TD group are on the (**right**).

**Figure 7:**
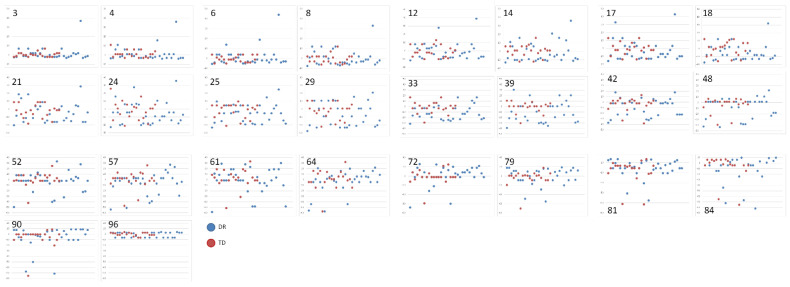
Scatter plots showing the mean EEs calculated for each of 26 numbers used in the 0–100 verbal task and for each participant. In each plot presenting the results obtained for a particular number magnitude, the dispersion revealed in the DR group is presented in blue, and that in the TD group is presented in red.

**Figure 8:**
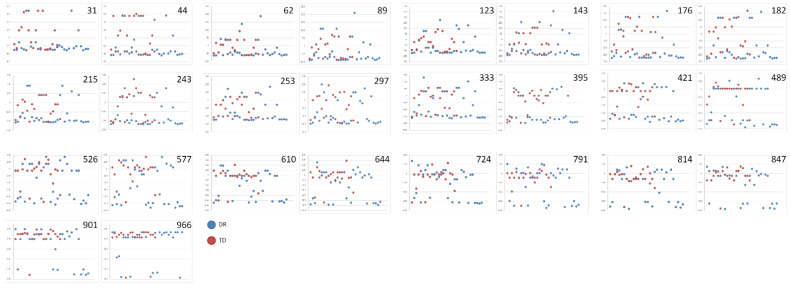
Scatter plots showing the mean EEs calculated for each of the 26 numbers used in the 0–1000 verbal task and for each participant. In each plot presenting the results obtained for a particular number magnitude, the dispersion revealed in the DR group is presented in blue, and that in the TD group is presented in red.

As a result, the mean group values do not demonstrate the actual level of NLE ability in the examined group of participants. This means that it is difficult to assess and make conclusions about a profile of its deficiency in children with dyscalculia or dyscalculia risk. Thus, instead of focusing on mean ratios, it is worth evaluating the individual differences in some examined capabilities by looking at the variability of individual results. However, in the case of the EE index obtained in the present study, both measures of central tendency (median value) and the measure of variability (e.g., IQR) are inadequate. Actually, an investigation regarding the individual data scatter seems to be more informative in the context of the group profile of ability, as presented in Figure 5, Figure 6, Figure 7 and Figure 8. The scatter plots concerning the results averaged from the whole stimuli set used in tasks 1 and 2 (Figure 5 and Figure 6), as well as the results calculated for each of the 26 numerical magnitudes (as presented in Figure 7 and Figure 8) show the comparisons of the two examined groups of children in regard to each condition.

In most of these plots, there are clear differences between the groups, which are visible in the individual data dispersion around the expected number value.

## 4. Discussion

We investigated the effect of dyscalculia risk on precision in the spatial localization of numbers on bounded empty number lines ranging from 0 to 100 and from 0 to 1000. The index of this precision level was the estimation error measured in two types of NLE tasks, in which the participants were asked (1) to verbally determine the number magnitude indicated on a line with an arrow and (2) to point to the place on a line related to the particular number magnitude displayed on a screen. This effect was analyzed not only in terms of the significance of the differences between the DR group and the TD control but also in terms of the variability of the individual performance data, which was revealed through descriptive statistics (here, the IQRs were calculated) and the scatter plots of the individual results within each group, both for the whole set of numbers and separately for each number used in the tasks.

First, for the verbal NLE task, a significant group difference, which was analyzed for the mean values obtained from all the numbers in the task, was found for the 0–1000 number line, while the difference between the DR and TD groups in the case of the 0–100 number line was insignificant. The descriptive statistics (Figure 3 and Table 1) revealed that, in the case of the 0–1000 number line, the variance in the data within the DR and TD groups was greater than in the case of the 0–100 number line. Moreover, in the verbal task using numbers from 0 to 100, the group median values were smaller than for the 0–1000 range, and the estimation error in the TD group had the opposite direction than that obtained in the DR group. The children with DR underestimated the number magnitude indicated at the given point of the number line much more frequently, while the control group overestimated it more frequently. Moreover, it is clearly illustrated in the scatter plots presenting the individual EEs in the groups (Figure 5 and Figure 6). It is probable, however, that all these variability characteristics found in the groups for these tasks led to no significant group differences (which indicates there was no effect of dyscalculia) in the 0–100 range and, at the same time, to a significant effect in the 0–1000 range.

A different pattern of results was found for the number-to-position tasks. In both numerical ranges, the participants with DR revealed greater EEs than the children in the TD group. It is worth emphasizing that the medians and ranges of values seem to suggest that all the children overestimated (i.e., they located numbers more or less to the right side of their appropriate place on the number line). However, this is not the case. The calculation method for the estimation error in this type of task (see Section 2.2 in Materials and Methods section) focuses on the absolute distance independent of the direction (measured in pixels) between the correct place of the number and the place indicated by the participant. This means that the value of EE can only be a positive number. This is a serious limitation of the methodology.

In addition, the variance (illustrated using IQR values; see Figure 4 and Table 2) was not similarly small. What is remarkable is that the variability was greater in the TD than in the DR group in the 0–100 task, while, in the 0–1000 task, the pattern was the opposite. However, although the group difference in the estimation errors for the 0–100 task was relatively smaller than it was for the 0–1000 task, this difference was statistically significant.

The different results obtained for the two types of NLE tasks (the verbal determination of number versus the spatial indication of number localization) indicate a need for the complex examination of spatial–numerical relationships using various methods. They also suggest that we must take into consideration the effects of the measurement method on the results and the possibility of comparing the obtained results with those from previous studies. This is because Skagerlund and Traff [41], for example, used the number-to-position tasks, while Ashcraft and Moore [29], for example, used the verbal type.

Furthermore, verbal tasks seem to be more demanding than number-to-position tasks, which is manifested in the high value of central tendency and variability statistics. We observed this difference in the difficulty between the tasks during the study (the participants reported difficulty).

Moreover, vast data variance in both the DR and TD groups suggests a serious general problem related to how mathematical abilities, such as mental number line processing, can be measured. Therefore, it would be unreasonable to compare our findings with those of other studies in which distinct methods were applied [51] or the examined groups differed markedly in terms of variance. This means that, in fact, all the NLE model curves described in the literature [29,32,52] for each numerical and age range may not apply. Usually, small and heterogeneous groups of participants with DD might consist of children with different subtypes of dyscalculia and, therefore, differ in terms of several numerical abilities, such as NLE.

An additional methodological limitation is the fact that, in many previous studies on DD, children were diagnosed (and included in the studies) using various types of examination methods [8]. Finally, the groups of participants varied in terms of several consequential factors that were not controlled in the studies (e.g., memory, attention, and executive functions).

Furthermore, methodological problems may also affect data collection. We encountered several such difficulties with the participants in our study. For example, some children with DR complained that they did not know any numbers greater than 100. Thus, performing the tasks with the 0–1000 number line was very difficult for them. As a result, they tried to guess the number magnitude corresponding to a particular place on the number line (in verbal tasks) or to guess the place corresponding to a given number magnitude (in the number-to-position task). In contrast, some TD children performed these tasks quite carelessly. One probable reason is that, for some of them, these tasks were effortless or even boring. Similar remarks were noted in a study by Gut et al. [34].

Additionally, we focused separately on each of the 26 numbers given in the verbal tasks. The results confirmed the conclusions drawn from the analyses completed for the whole set of numbers (the means calculated for all the numbers). The highest IQR values were mainly those for the number magnitude around the center of the 0–100 number line, especially in the DR group. This suggests that the children with DR revealed a meaningful problem with setting a benchmark in the center of a given number line to indicate the correct number in the given locus. This seems to be the result, which is typical for much younger, healthy children. In addition, children with DR generally underestimated nearly all the numbers on the 0–100 line, except for some magnitudes close to the center and those in the right half of the number line (numbers: 48, 64, 72, 79, 96). In the TD children, almost all the median values show overestimation; however, they are very low and close to zero. Despite relatively high values of estimation error in the DR group, we obtained the significance effect of the group factor for only two numbers: 18 and 33. None of them are located close to the center of the number line. The insignificance of group differences for almost all the number values was due to the huge variability in the data, especially in the DR group.

The results revealed that the 0–1000 number line presented a completely different pattern of group differences. The differences between the groups were statistically significant for 13 of the 26 number magnitudes; however, none of these values were concentrated around the center of the line. The lack of significant differences for numbers located close to the center may mean that estimation error values in this range are similarly large in all children; however, this is not confirmed by the huge median values obtained in the DR group in comparison to the TD one (e.g., these obtained for numbers 333 or 395). Moreover, in the DR group, the median statistics were solely underestimations, which is consistent with the considerable effort required to process spatial representations of three-digit numbers observed in many children with DR.

The most impressive confirmation of heterogeneity in both groups, especially in the children with DR, was revealed in the scatter plots, which clearly illustrate that the participants differed not only in their estimation error values (which varied from very low to very high values) but also in the direction of their errors (i.e., some children underestimated, while others overestimated).

## 5. Conclusions

Therefore, the high variability within each group of participants and the differences between the extents of this variability for different tasks and particular numbers confront us with a very complicated picture of NLE task performance. In fact, there was almost no consistency in the NLE task performance, especially in the DR group. We can conclude that the significance in the differences between the mean or median values tells us nothing about the examined effects of a deficit until we focus on the data variance, heterogeneity of the group, and the impact of additional variables. This finding has important implications for research and diagnostic purposes.

It could even be concluded that, the greater the variability in one of the groups or in a whole examined sample, the more probable it is for the effect of the deficit (the difference between the groups) to be vague or absent. The variance obtained in the NLE performance may suggest that the similar problem concerns other numerical abilities as it was demonstrated—e.g., in the study concerning the SNARC effect.

Moreover, in future studies, it would be worth considering such factors as a subtype of dyscalculia and its coexistence with attentional deficits. However, there is no consistency in the way DD subtypes have been classified. These subtypes have been defined on the basis of some distinct theories and in accordance with different methods, several numerical abilities, and the combination of additional variables included in the analyses. All this leads to the requirement of using larger groups of participants or a larger number of DD groups (with regard to DD subtypes), which may be difficult to achieve given the fact that the DD population is very limited.

## Figures and Tables

**Table 1:** Descriptive statistics (median, IQR, and range) calculated for all numbers used in tasks 1 and 2 (0–100 and 0–1000, respectively) obtained in the DR and TD groups.

EE Obtained for Whole Task	DR Group (*n* = 32)			TD Group (*n* = 20)			*Z*	*p*
	*Me*	*IQR*	*Range*	*Me*	*IQR*	*Range*		
0–100 (task 1)	−3.42	10.08	−29.0–24.19	0.73	4.5	−31.35–6.58	ns	
0–1000 (task 2)	−138.73	286.98	−384.38–69.0	−12.77	132.88	−360.23–43.81	−2.085	0.037

**Table 2:** Descriptive statistics (median, IQR, and range) calculated for all numbers used in tasks 3 and 4 (0–100 and 0–1000, respectively) obtained in the DR and TD groups.

EE Obtained for Whole Task	DR Group (*n* = 32)			TD Group (*n* = 20)			*Z*	*p*
	*Me*	*IQR*	*Range*	*Me*	*IQR*	*Range*		
0–100 (task 3)	8.06	5.19	3.65–22.24	5.68	2.42	3.09–18.45	−2.69	0.007
0–1000 (task 4)	5.84	5.51	4.04–25.06	15.37	17.01	3.99–38.74	−3.762	<0.001

**Table 3:** Descriptive statistics (median, IQR, and range) calculated for each particular number used in task 1 (0–100 number line) obtained in the DR and TD groups.

Number Magnitude	DR Group (*n* = 32)			TD Group (*n* = 20)			*Z*	*p*
	*Me*	*IQR*	*Range*	*Me*	*IQR*	*Range*		
3	−1.0	4.0	−3.0–37	0	4.0	−2.0–7.0	ns	
4	−1.0	4.0	−4.0–36	1.0	6.0	−3.0–11.0	ns	
6	−2.0	8.0	−5.5–44.0	−1.0	7.0	−5.0–4.0	ns	
8	−2.5	7.0	−7.5–33.0	1.0	6.0	−6.0–12.0	ns	
12	−2.5	11.5	−11.5–39	−2.0	15.0	−10.0–13.0	ns	
14	−7.0	16.0	−13.0–36	1.0	10.0	−12.0–16.0	ns	
17	−7.0	15.75	−16.0–43.0	−2.0	6.0	−14.0–13.0	ns	
18	−8.0	14.5	−17.5–32.0	2.0	15.0	−16.0–12.0	−1.966	0.049
21	−6.0	19.75	−20.0–29.0	−1.0	16.0	−18.0–9.0	ns	
24	−8.0	24	−22.5–36	1.0	15.0	−21.0–26.0	ns	
25	−5.0	20.0	−23.0–25.0	2.0	10.0	−21.0–10.0	ns	
29	−9.0	31.0	−27.5–21.0	1.0	14.0	−22.0–11.0	ns	
33	−13.0	19.13	−31.0–17.0	−3.0	12.0	−29.0–17.0	−2.386	0.017
39	−9.0	27.5	−38.5–31.0	1.0	7.0	−30.0–11.0	ns	
42	−2.0	24.25	−37.0–18.0	−2.0	9.0	−38.0–13.0	ns	
48	2.0	23.75	−43.0–22.0	2.0	7.0	−38.0–7.0	ns	
52	−2.0	13.25	−49.5–33.0	−2.0	10.0	−42.0–25.0	ns	
57	−2.0	13.75	−54.0–27.0	3.0	14.0	−47.0–26.0	ns	
61	−1.0	27.75	−58.0–30.0	4.0	17.0	−51.0–33.0	ns	
64	1.5	12.5	−57.0–22.0	1.0	18.0	−57.0–32.0	ns	
72	7.0	18.75	−68.0–26.0	−2.0	5.0	−59.0–25.0	ns	
79	7.00	20.75	−55.0–19.0	1.0	11.0	−70.0–18.0	ns	
81	−1.0	14.5	−65.0–17.0	2.0	15.0	−73.0–17.0	ns	
84	−0.5	24.0	−82.0–15.0	−2.0	10.0	−75.0–13.0	ns	
90	0	17.75	−71.0–9.0	0	10.0	−75.0–9.0	ns	
96	3.0	9.0	−6.0–4.0	0	4.0	−6.0–4.0	ns	

**Table 4:** Descriptive statistics (median, IQR, and range) calculated for each particular number used in task 2 (0–1000 number line) obtained in the DR and TD groups.

Number Magnitude	DR Group (*n* = 32)			TD Group (*n* = 20)			*Z*	*p*
	*Me*	*IQR*	*Range*	*Me*	*IQR*	*Range*		
31	−26.0	11.5	−31.0–69.0	−16.0	46.0	−31.0–69.0	ns	
44	−34.0	32.0	−44.0–57.0	−14.0	92.0	−43.0–61.0	−2.023	0.043
62	−50.0	32.5	−61.5–188.0	−12.0	90.0	−59.0–93.0	−2.02	0.043
89	−72.5	49.5	−88.5–211.0	−19.0	125.0	−86.0–71.0	ns	
123	−103.0	106.25	−122.0–177.0	−23.0	105.0	−118.0–92.0	−2.1	0.036
143	−110.5	168.75	−141.0–257.0	−43.0	180.0	−135.0–107.0	ns	
176	−150.5	130.5	−173.0–166.0	−16.0	190.0	−171.0–124.0	−2.543	0.011
182	−142.0	90.0	−178.0–160.0	−52.0	195.0	−172.0–168.0	−2.251	0.024
215	−187.5	92.5	−212.0–185.0	−85.0	188.0	−206.0–85.0	−2.024	0.043
243	−203.0	182.5	−238.0–257.0	−43.0	271.0	−233.0–157.0	−2.1	0.036
253	−207.0	185.0	−248.0–175.0	−103.0	213.0	−240.0–97.0	−1.997	0.046
297	−244.0	266.0	−292.0–203.0	−57.0	302.0	−282.0–192.0	−2.325	0.020
333	−277.0	351.75	−325.0–267.0	2.0	320.0	−303.0–117.0	−2.148	0.032
395	−315.0	375.0	−390–133.0	5.0	335.0	−386.0–87.0	ns	
421	−331.0	350.0	−409.0–79.0	−21.0	152.0	−351.0–77.0	−2.572	0.01
489	−39.0	444.5	−477.0–137.0	11.0	206.0	−439.0–87.0	ns	
526	−227.5	525.0	−514.0–174.0	−26.0	240.0	−476.0–142.0	ns	
577	−322.5	530.0	−557.0–173.0	23.0	128.0	−477.0–173.0	−2.548	0.011
610	−110.0	496.0	−543.0–194.0	−10.0	73.0	−530.0–206.0	−2.134	0.033
644	−79.5	607.75	−590.0–256.0	−3.0	120.0	−544.0–286.0	ns	
724	−74.0	635.75	−647.0–274.0	−24.0	130.0	−624.0–230.0	ns	
791	−91.0	665.25	−741.0–208.0	−21.0	126.0	−696.0–182.0	ns	
814	−114.0	634.0	−764.0–136.0	−25.0	120.0	−724.0–161.0	ns	
847	−47.0	637.0	−767.0–149.0	−47.0	200.0	−757.0–152.0	ns	
901	−101.0	604.5	−812.0–98.0	−1.0	115.0	−811.00–89.0	ns	
966	−37.0	379.5	−867.0–34.0	−16.0	92.0	−866.0–33.0	ns	

## Data Availability

Data are available from authors upon request.
